# Cytological Performance Evaluation of a Novel Skin-Repair Agent: Recombinant Protein Disulfide Bond Isomerase

**DOI:** 10.3390/bioengineering13091032

**Published:** 2026-09-05

**Authors:** Xin Luo, Qirong Zhang, Wantong Liu, Dan Li, Huawei Zhang, Xiaobin Yang, Jianhang Cong, Shu Zhang, Yating Cheng, Qi Xiang

**Affiliations:** 1 State Key Laboratory of Bioactive Molecules and Druggability Assessment, Jinan University, Guangzhou 510632, China; luoxin@stu2023.jnu.edu.cn (X.L.); qi360@stu2025.jnu.edu.cn (Q.Z.); liuwantong@stu2023.jnu.edu.cn (W.L.); 2112440212@stu.gdpu.edu.cn (D.L.); 2112542177@stu.gdpu.edu.cn (H.Z.); yxbpost@163.com (X.Y.); 2022c@stu2022.jnu.edu.cn (J.C.); 2 Guangdong Provincial Key Laboratory for Research and Evaluation of Pharmaceutical Preparations, Guangdong Pharmaceutical University, Guangzhou 510006, China; zhangshu@gdpu.edu.cn; 3 Institute of Cosmetics and Medical Aesthetics, Jinan University, Guangzhou 510632, China; 4 Biopharmaceutical Research & Development Center of Jinan University, Guangzhou 510632, China

**Keywords:** recombinant protein disulfide isomerase, skin repair, cell adhesion, cosmetic raw material

## Abstract

To investigate the potential of recombinant protein disulfide isomerase (PDI) as a raw material for skin-repair cosmetics, an engineered PDI-producing strain was constructed using the *Pichia pastoris* X33 expression system. The target protein was obtained through methanol-induced expression and anion-exchange chromatography purification, and its cytotoxicity and pro-adhesive effects on human skin fibroblasts (HSFs) were evaluated. The results showed that the purified recombinant PDI protein had an apparent molecular weight of approximately 55.7 kDa. Treatment with 0.04–5 µmol/L recombinant PDI for 24 h did not affect cell viability. On culture surfaces coated with 62.5 nmol/L recombinant human fibronectin (rhFN) or 31.25 nmol/L recombinant human collagen (rhC), the addition of 250 nmol/L recombinant PDI increased the HSF adhesion area ratio to approximately 2.1-fold that of the rhFN-alone group (*p* < 0.0001) and 1.9-fold that of the rhC-alone group (*p* < 0.0001). Collectively, the recombinant PDI protein exhibits good biocompatibility and pro-adhesive activity, demonstrating its potential as a candidate raw material for skin-repair cosmetics.

## 1. Introduction

As the largest organ of the human body, the skin serves as the primary barrier against external physical, chemical, and microbial damage, playing a pivotal role in maintaining internal environmental stability, regulating immune responses, and sensing external stimuli [1]. With advancements in research on skin repair, anti-aging, and functional cosmetics, preserving skin structural integrity, improving the microenvironment for post-injury repair, and promoting skin function recovery have become key focuses in skin biology and cosmetic ingredient development. Skin health not only depends on the integrity of the epidermal barrier function but also closely relates to the normal synthesis, folding, secretion, and orderly extracellular assembly of proteins within the dermal extracellular matrix (ECM). Among these, extracellular matrix components such as collagen (COL) and fibronectin (FN) are essential for maintaining mechanical support and tissue structural stability; their proper processing and assembly are critical for regulating fibroblast adhesion, migration, proliferation, and wound healing [2,3,4]. COL provides structural scaffolding and integrin-mediated signaling; FN, via RGD motifs, mediates adhesion and migration. Plasma FN forms a provisional matrix, while cellular FN later coordinates with COL to build mature ECM and guide tissue remodeling.

When the skin is subjected to factors such as trauma, inflammation, ultraviolet radiation, or aging, intracellular levels of oxidative stress increase, making the protein homeostasis of the endoplasmic reticulum susceptible to disruption [5,6]. Secretory ECM-associated proteins destined for secretion (e.g., COL and FN) typically require initial folding, disulfide bond formation, and partial post-translational modifications within the endoplasmic reticulum before assuming their functional roles. Subsequently, they undergo further processing and secretion by the Golgi apparatus, followed by maturation, deposition, and assembly outside the cell [5,6,7]. When endoplasmic reticulum folding capacity declines or the redox microenvironment becomes imbalanced, unfolded or misfolded proteins can accumulate within the reticulum, triggering endoplasmic reticulum stress and activating the Unfolded Protein Response (UPR) [5,6]. While moderate UPR contributes to restoring protein homeostasis, persistent stress or exceeding cellular adaptive capacity—defined as the ability of cells to sense and respond to environmental or metabolic stresses by adjusting gene expression, protein folding, and redox homeostasis to maintain survival and function—may impair the maturation, secretion, and extracellular assembly of ECM-associated proteins, reduce skin cell viability, and disrupt adhesion, migration, and proliferation between fibroblasts and the ECM, ultimately hindering skin injury repair. This process may also contribute to pathological conditions such as skin aging, chronic wound non-healing, and photoaging [8,9]. Therefore, elucidating the relationship between endoplasmic reticulum-mediated protein-folding quality control and ECM remodeling during skin repair, as well as developing functional molecules that mitigate protein-folding abnormalities and improve the ECM repair microenvironment, holds significant research value.

The PDI is a key enzyme within the thioredoxin superfamily, responsible for catalyzing the formation, cleavage, and rearrangement of disulfide bonds. Primarily localized in the endoplasmic reticulum lumen, it promotes proper folding of secretory and membrane proteins and inhibits misfolded protein aggregation through molecular chaperone function, thereby maintaining protein homeostasis [10]. During the formation of skin-associated ECM, PDI not only participates in quality control of COL and FN—the two major structural proteins of the dermal matrix—but also functions as the β-subunit of collagen prolyl-4-hydroxylase (P4H) in the maturation of procollagen, playing a critical role in stabilizing collagen fibrils [11,12,13]. Additionally, certain members of the PDI family are localized to the cell surface or extracellular environment, where they modulate protein thiol groups and disulfide bond interactions to influence extracellular protein conformation and cell adhesion processes [14,15]. The ERp57 protein encoded by the PDIA3 gene, a member of the PDI family, has been shown to participate in FN-mediated matrix formation and fibroblast adhesion, facilitating FN-related matrix assembly and cell spreading [16,17]. Notably, Weiß et al. demonstrated that human PDI (hPDI) is natively unglycosylated, a finding consistent with our bioinformatics predictions [18]. Therefore, the recombinant hPDI expressed in the Pichia pastoris system in this study is expected to be free of glycan-related heterogeneity, eliminating potential interference in subsequent functional assays. Thus, PDI not only regulates intracellular protein folding homeostasis but also potentially influences the conformational maintenance, matrix assembly, and cell–matrix adhesion of ECM proteins such as collagen and FN, thereby exerting regulatory roles in skin repair and barrier function restoration.

During skin injury or aging, elevated reactive oxygen species (ROS) disrupt cellular redox balance and ER protein homeostasis—conditions that are closely linked to PDI function [10,14]. Therefore, exogenous supplementation with active recombinant PDI proteins may help improve local protein-folding homeostasis, enhance cell–matrix adhesion, and provide novel insights for skin functional repair.

Naturally occurring PDI typically requires isolation and purification from animal tissues or other biomaterials, involving relatively complex preparation procedures with limitations regarding source stability, batch consistency, and subsequent application development [19,20]. In addition, animal-derived materials may carry xenogeneic antigens and potential animal pathogens, creating risks of immune responses and pathogen transmission. Clinical studies of bovine collagen implants have documented anti-collagen antibodies and localized adverse reactions, whereas porcine dermal collagen membranes have also elicited humoral responses that vary with processing conditions [21,22]. Genetic engineering provides a viable approach for the efficient production of recombinant PDI, where the Pichia pastoris expression system combines advantages such as eukaryotic expression, secretory expression, and large-scale production, making it suitable for recombinant protein synthesis and optimization [23]. Notably, PDI family members such as AGR2 and PDIA3/ERp57 have been systematically evaluated in skin-repair models, demonstrating pro-migratory and pro-adhesive activities [16,24]. Currently, systematic studies on the functional role of recombinant PDI in skin biology models—particularly its impact on the adhesion behavior of dermal fibroblasts—remain scarce.

To facilitate efficient production and purification of recombinant PDI, we adopted the widely used P. pastoris secretion system with the pPICZαA vector. The pPICZαA vector, featuring an α-factor signal peptide, is commonly used in P. pastoris secretion systems to direct recombinant protein export into the culture supernatant, thereby facilitating downstream purification. This system also supports high-density cultivation and industrial-scale production, and it remains widely adopted for recombinant protein expression [25].

Fibroblasts are commonly used as a model for evaluating the cellular compatibility of candidate materials for skin regeneration [26]. Based on these technical advantages, this study utilized the Pichia pastoris expression system to establish and optimize the expression and purification protocol for recombinant PDI proteins, yielding high-purity, bioactive recombinant PDI. Subsequently, through cytotoxicity assays and cell adhesion experiments conducted on interfaces coated with rhFN and rhC, we systematically evaluated the compatibility and adhesion-promoting effects of recombinant PDI on HSFs, thereby assessing its potential applications in cosmetic skin repair and anti-aging therapies.

## 2. Materials and Methods

### 2.1. Material

#### 2.1.1. Strains and Plasmids

The strains used, Pichia pastoris X33 and Escherichia coli Top10, were stored in our laboratory. The vector employed was pPICZαA, also stored in our laboratory. All samples were aliquoted and preserved at –80 °C to prevent repeated freeze–thaw cycles. The PDI protein gene (GenBank Accession No.: 5034; XM_024450777.1) was synthesized by GenScript Biotech Inc. (Nanjing, China). HSFs were maintained in our laboratory.

#### 2.1.2. Main Reagents, Materials, and Instruments

The restriction enzymes XbaI, EcoRI, SacI, and T4 DNA ligase were purchased from TaKara Bio; the plasmid extraction kit was obtained from Beijing Tiangen Biochemical Technology Co., Ltd. (Beijing, China); high-fidelity PCR enzymes were supplied by TaKara Bio; the DNA Marker 5000 was provided by TaKara Bio; recombinant fibronectin (rhFN) and recombinant collagen (rhC) were constructed and purified in our laboratory [27,28]. LB medium: 0.5% (m/V) yeast extract; 1% (m/V) peptone; 1% (m/V) sodium chloride; YPD: 1% (m/V) yeast extract; 2% (m/V) peptone; 2% (m/V) glucose; YPG: 1% (m/V) yeast extract; 2% (m/V) peptone; 2% (V/V) glycerol; YPM: 1% (m/V) yeast extract; 2% (m/V) peptone; 1% (V/V) methanol; 1 mol/L sorbitol; LiAc-DTT solution. All culture media and reagents were sterilized by autoclaving at 120 °C for 20 min.

The RealBand pre-stained protein markers (14.4–116 kDa), SDS-PAGE kit, primary antibody dilution buffer, and blocking solution were provided by Shanghai Biyuntian Company (Shanghai, China). The PDIA2 rabbit polyclonal antibody (A12789) was purchased from AbClonal Company (Wuhan, China). The HRP-labeled goat anti-rabbit IgG was obtained from Yeason (Shanghai, China). The BCA protein assay kit was supplied by Sigma Scientific Corporation of the United States (St. Louis, MI, USA). Tris-base was obtained from Thermo Fisher Scientific Corporation of the United States (Waltham, MA, USA). HSFs were stored in our laboratory; all other unlabeled reagents were domestic, analytical-grade products.

The high-pressure sterilizer (HVE-50) was purchased from HIRAYAMA Co., Ltd. (Tokyo, Japan) in Japan; the electrophoretic turner (165-2100) from Bio-Rad Inc. (Hercules, CA, USA) in the United States; the chemiluminescence image analyzer (Tanon 4200SF) from Shanghai Tianneng Technology Co., Ltd. (Shanghai, China); the ultrapure water generation system (Milli-Q model) from Millipore; the constant-temperature incubator–shaker from Jiangsu Qilin Bell Instrument Co., Ltd. (Jiangsu, China); the protein electrophoresis instrument from Bio-Rad Inc. in the United States; the semi-dry membrane transfer system from Bio-Rad Inc. in the United States; the electronic balance (BT 125D) from Sartorius (Göttingen, Germany); the low-speed centrifuge from Shanghai Anting Scientific Instrument Factory (Shanghai, China); the clean workbench (SW-CJ-2FD) from Suzhou Antai Air Technology Co., Ltd. (Suzhou, China); the full-wavelength microplate reader from Thermo Fisher Scientific Inc. (Waltham, MA, USA) in the United States; the fluorescence inverted microscope from Olympus; and the CO_2_ incubator (BPN-150RHP) from Shanghai Yiheng Scientific Instrument Co., Ltd. (Shanghai, China).

### 2.2. Methods

#### 2.2.1. Construction of the Recombinant Expression Vector pPICZαA-PDI

Based on the gene sequence of human PDI (GenBank Accession No.: 5034; XM_024450777.1), Nanjing GenScript Biotechnology Co., Ltd (Nanjing, China). was commissioned to perform full-genome synthesis. Using this synthesized gene as a template, the PDI gene sequence was amplified via specific primers and a PCR protocol, with restriction sites XbaI and EcoRI introduced. The double-cut products were then ligated into the pPICZαA plasmid to form the pPICZαA-PDI plasmid. The reaction product was transformed into competent Escherichia coli Top10 cells, positive strains were screened, plasmids were extracted, and sequencing was performed for identification after restriction enzyme digestion verification. The primer sequences used in the experiment are listed in Table 1.

#### 2.2.2. Preparation of Competent Cells of Saccharomyces Cerevisiae X33, Electroporation, and PCR Identification of Positive Transformants

Using an electroporation device (Bio-Rad,USA), 10 µL of the recombinant plasmid pPICZαA-PDI linearized with Sal I was transferred into 100 µL of prepared competent X33 yeast cells, pre-cooled on ice for 5 min, and immediately subjected to electroporation under the following conditions: 1.5 kV, 50 µF capacitance, and 200 Ω resistance. Immediately after electroporation, 1 mL of ice-cold sorbitol solution $\left(1.0\frac{mol}{L}\right)$ was added, and the mixture was incubated under shaking at 30 °C for 1 h. Subsequently, 100 µL of the bacterial suspension was spread onto YPD plates containing 2.0 mg/mL Zeocin antibiotic and cultured at 30 °C for 2 days to screen for positive transformants with high copy numbers.

#### 2.2.3. Purification of Recombinant PDI Protein

Select single colonies with high copy numbers and inoculate them into 5 mL of YPD culture medium, and then incubate at 28 °C for 24 h under 220 rpm. Centrifuge the culture broth, remove the supernatant, transfer the bacterial cells to 5 mL of YPG culture medium, and continue incubation at 28 °C and 220 rpm. When the cell OD_600_ reaches 2–8, collect the bacterial suspension, resuspend it until achieving an OD_600_ of 1.0, and transfer it to YPM culture medium for induction of PDI protein expression. Sample the culture every 24 h and add methanol to the YPG medium to achieve a final concentration of 1%. Collect samples and centrifuge them at 12,000 rpm for 3 min at room temperature; collect the supernatant and determine PDI expression levels by SDS-PAGE.

#### 2.2.4. Induced Expression of the Recombinant Bacterial Strain

After the induction expression was completed, the fermentation supernatant was collected as the sample for subsequent purification. Based on the theoretical isoelectric point of the target protein PDI being approximately 4.8, Q Sepharose Fast Flow anion-exchange chromatography was performed under pH 8.0 conditions. Elution buffers were prepared using 20 mmol/L Tris-base buffer (pH 8.0) as the base, containing 100, 300, and 600 mmol/L, and 1 mol/L NaCl, respectively. Prior to loading, the Q column was washed with 0.5 mol/L NaOH for 3–5 column volumes, followed by rinsing with ultrapure water until neutralization; the column was then balanced with 100 mmol/L NaCl-Tris-base buffer until baseline stability was achieved. The induced culture supernatant was filtered through 0.80 µm, 0.45 µm, and 0.22 µm membranes in a suction filtration flask to remove bacteria, and the pH was adjusted to 8.0 before loading, with each loading volume approximately 200 mL. After loading, gradient elution was performed using Tris-base buffer with varying NaCl concentrations, and the permeate along with each elution fraction was collected sequentially. Upon completion of chromatography, the column was washed with 0.5 mol/L NaOH for 3–5 column volumes and rinsed with ultrapure water until neutralized for further use.

#### 2.2.5. Western Blot Analysis of Recombinant PDI Protein

After separation of the purified target protein by SDS-PAGE, transfer it to a PVDF membrane (200 mA, 90 min). Block the membrane with blocking buffer at room temperature for 1.5 h, and then add primary antibody (PDIA_2_ Rabbit pAb, diluted 1:3000) and incubate overnight at 4 °C. Wash the membrane three times with TBST, followed by the addition of secondary antibody (HRP-labeled goat anti-rabbit IgG, diluted 1:10,000) and incubation at room temperature for 1 h. Wash again three times with TBST, develop the gel using ECL under light protection for 1 min, and observe the results on a gel imaging system.

#### 2.2.6. Detection of Cytotoxicity of Recombinant PDI Protein Against HSF Cells

HSF cells in the logarithmic growth phase were seeded at 1 × 10^4^ cells per well in a 96-well plate. When the cell density reached 70–80%, different concentrations of recombinant PDI protein (0.04, 0.2, 1, and 5 µmol/L) were added and incubated for 24 h. Each group included five replicate wells, along with a solvent control group and a blank group. The supernatants were removed, and each well was supplemented with serum-free medium containing 10% CCK-8 for further incubation for 2 h. Absorbance was measured at 450 nm.

Cell survival rate calculation formula: (Experimental group − Control group)/(Control group − Blank group) × 100%.

#### 2.2.7. Cell Adhesion Assay Using Recombinant PDI Combined with rhC or rhFN

The 96-well plates were coated with 62.5 nM rhFN and 31.25 nM rhC, respectively. HSF cells were then seeded at a density of 8 × 10^3^ cells/100 µL and incubated together with varying concentrations of recombinant PDI protein (2, 10, 50, and 250 nmol/L). After 5 h, unattached cells were washed off with PBS. Cells were fixed with 4% polyformaldehyde for 20 min, rinsed with PBS 1–2 times, stained with 100 µL of 0.1% crystal violet for 30 min, and rinsed again with PBS 1–2 times. Images were observed under an inverted fluorescence microscope, and the proportion of cell adhesion area was calculated using the analysis software ImageJ (version 2.0.0) to evaluate the effect of recombinant PDI protein on HSF cell adhesion capacity.

#### 2.2.8. Statistical Analysis

Statistical analysis was performed using GraphPad Prism 9.5.1, with each experiment involving five parallel replicate groups. All data are presented as the mean ± standard deviation.

## 3. Results and Discussion

### 3.1. Construction of the Recombinant Expression Vector pPICZαA-PDI and Screening of Cloned Strains

**Figure 1:**
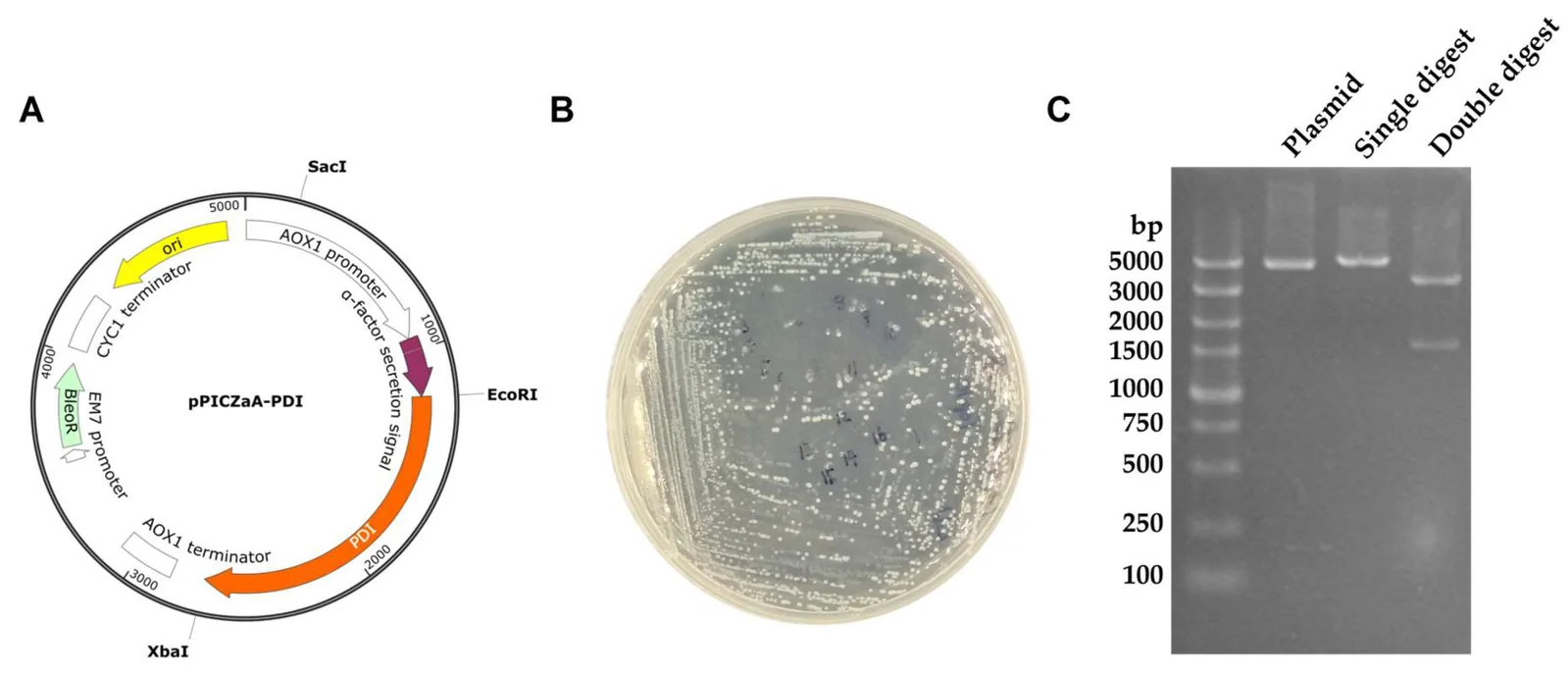
Construction of the recombinant expression vector pPICZαA-PDI: (**A**) Recombinant pPICZαA-PDI plasmid. (**B**) Transformation of Escherichia coli Top10 with the recombinant pPICZαA-PDI. (**C**) Identification of positive clones of recombinant pPICZαA-PDI by restriction enzyme digestion and nucleic acid electrophoresis. M: DL5000 DNA Marker.

To obtain recombinant PDI proteins suitable for functional evaluation, we first constructed the *Pichia pastoris* expression vector pPICZαA-PDI. The codon-optimized PDI gene fragment was cloned into the pPICZαA vector, yielding the recombinant plasmid pPICZαA-PDI (Figure 1A). After transforming *E. coli* Top10 competent cells with the recombinant plasmid, white single colonies were observed on LB plates supplemented with bleomycin (Zeocin) (Figure 1B). Plasmids were extracted from positive clones and subjected to restriction enzyme digestion analysis; agarose gel electrophoresis results (Figure 1C) demonstrated that single restriction digestion produced a band of approximately 5100 bp, consistent with the theoretical plasmid size (5131 bp), while double digestion yielded a target gene fragment of ~1600 bp and a vector backbone fragment of ~3500 bp, both matching the expected sizes. These findings confirm successful insertion of the PDI gene into the pPICZαA vector and the successful construction of the recombinant expression vector pPICZαA-PDI, which is suitable for subsequent Pichia pastoris transformation.

### 3.2. Screening of Expression Bacteria for the Recombinant Delivery Vector pPICZαA-PDI

**Figure 2:**
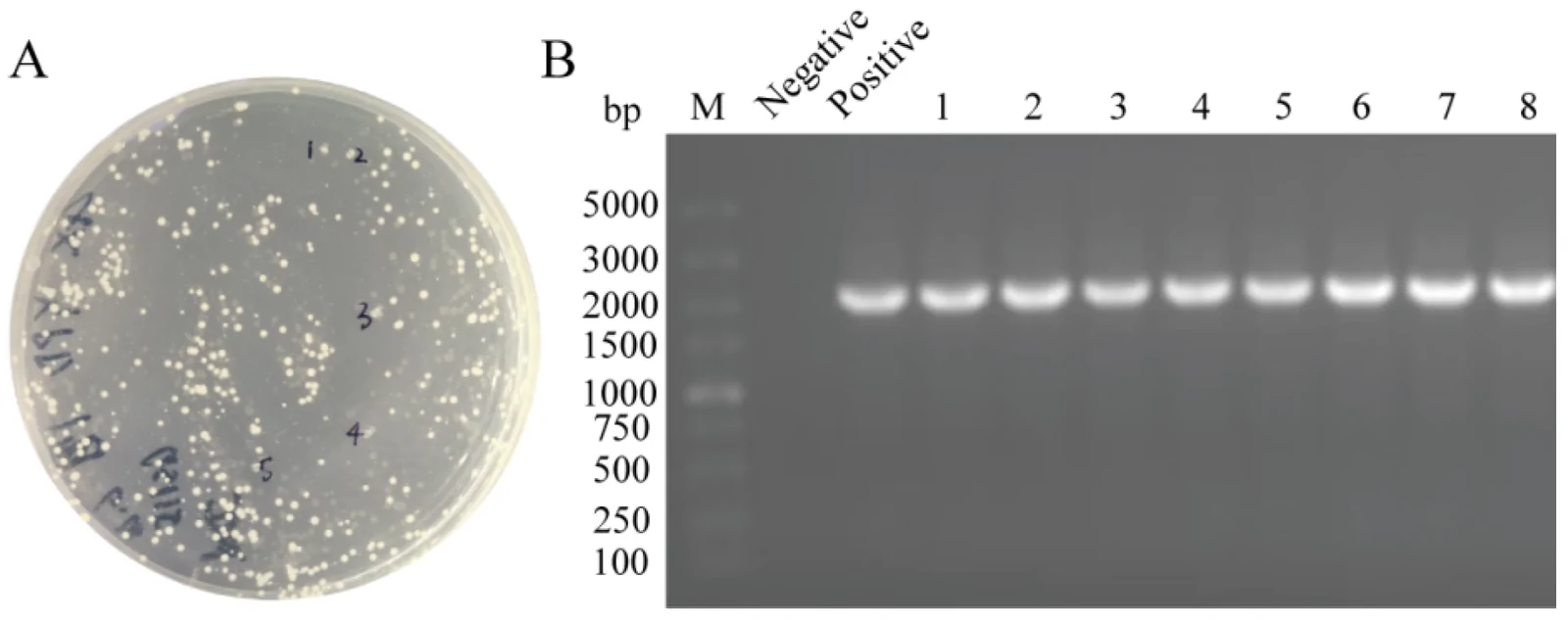
Screening of expression host for the recombinant expression vector pPICZαA-PDI: (**A**) Transformation of Pichia pastoris (X33) with the recombinant pPICZαA-PDI. (**B**) Identification of positive single colonies by nucleic acid.

To obtain an engineered Pichia pastoris strain capable of stable PDI expression, after sequence verification through sequencing, the linearized recombinant plasmid was electroporated into competent Pichia pastoris X33 cells and screened using Zeocin resistance plates. After 2–3 days of culture, distinct single colonies were observed on the plates (Figure 2A), indicating that the transformed strains exhibited the corresponding resistance phenotype. Fifteen randomly selected colonies from YPD resistance plates were subjected to colony PCR analysis, with the recombinant plasmid pPICZαA-PDI serving as the positive control and untransformed X33 yeast colonies as the negative control. PCR products were analyzed by 1% agarose gel electrophoresis, with the results shown in Figure 2B. All 15 transformants produced specific bands consistent with those from the positive control, approximately 2200 bp in size, matching the length of the target PDI gene fragment, whereas no amplification bands were detected in the negative controls. These results demonstrate that the recombinant expression vector pPICZαA-PDI was successfully integrated into the Pichia pastoris X33 genome and is suitable for subsequent induction expression experiments.

### 3.3. Induced Expression, Purification, and Western Blot Identification Results of Recombinant PDI Protein

**Figure 3:**
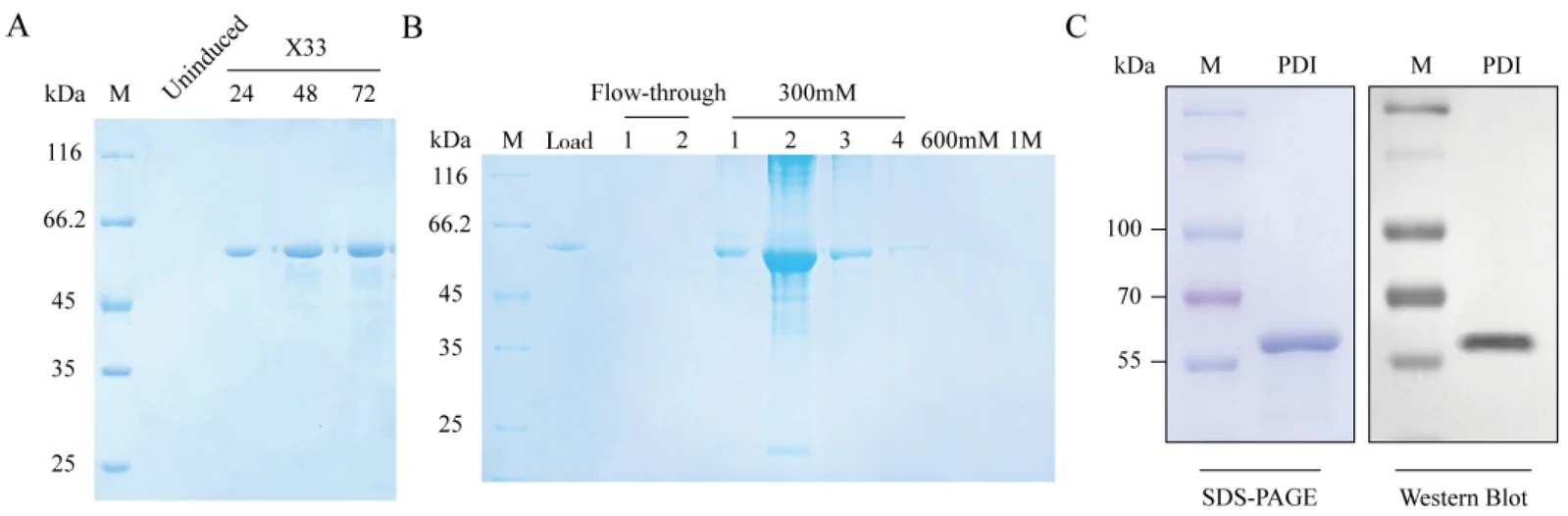
Induced expression (**A**), purification (**B**), and SDS-PAGE and Western blot analysis (**C**) of the recombinant PDI protein.

After obtaining positive transformants, this study further evaluated the expression of recombinant PDI protein using methanol induction. As shown in Figure 3A, the recombinant PDI protein displayed distinct bands in the range of 45 to 66.2 kDa from 24 to 72 h following induction, as determined by SDS-PAGE. This finding was consistent with the size of recombinant PDI protein (approximately 55.7 kDa). Cultivation time significantly influenced protein expression, with the highest expression levels observed after 72 h of induction. Following induction, recombinant PDI protein was purified using a Q Sepharose Fast Flow anion-exchange column. Since the engineered bacteria produced soluble recombinant protein, the treated supernatant was directly subjected to natural purification. The analysis in Figure 3B demonstrates the presence of highly pure target protein in an elution buffer containing 300 mmol/L NaCl and 20 mM Tris base. Western blot analysis of the soluble recombinant protein by SDS-PAGE confirmed that the purified protein was indeed PDI (Figure 3C).

This study confirmed the successful expression and preliminary purification of recombinant PDI via SDS-PAGE and Western blot, providing essential experimental materials for subsequent functional evaluation in cells.

### 3.4. Cytotoxicity Evaluation of Recombinant PDI Protein

**Figure 4:**
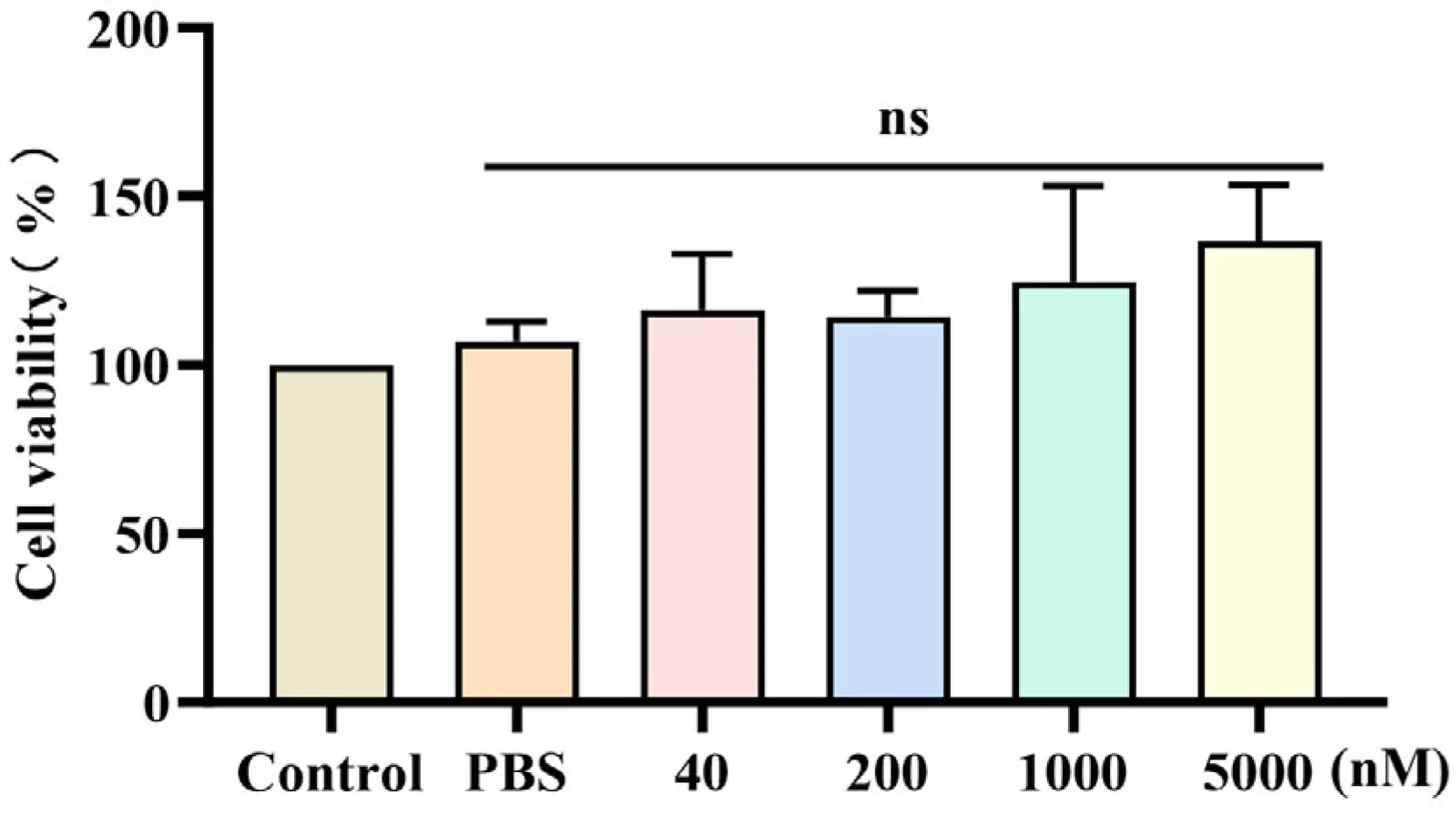
Effects of different concentrations of recombinant PDI protein on the growth of HSF cells; *n* = 5. Data are expressed as mean ± SD and were analyzed using one-way ANOVA; ns: no significant difference compared with the control group.

To assess the fundamental cellular compatibility of recombinant PDI protein with HSFs, this study employed the CCK-8 assay to measure changes in HSF cell viability after 24 h of exposure to different concentrations of recombinant PDI. The results demonstrated that, compared to the control group, no significant reduction in cell survival was observed in the treatment groups exposed to 0.04, 0.2, 1, or 5 µmol/L recombinant PDI; no statistically significant differences were noted between any treatment group and the control group (Figure 4). These findings indicate that, within the concentration range and exposure duration tested, recombinant PDI exhibits no notable cytotoxicity toward HSF cells and demonstrates favorable short-term in vitro cellular compatibility.

Good cellular compatibility serves as the foundation for conducting subsequent functional evaluations of protein-based candidates as skin-repair ingredients. The aforementioned results demonstrate that recombinant PDI exhibits no adverse effects on the short-term viability of HSF cells within the concentration range of 0.04–5 µmol/L, providing experimental evidence for further assessment of its role in fibroblast adhesion-related functions.

### 3.5. The Effect of Recombinant PDI Combined with rhC or rhFN on Cell Adhesion Capacity

Fibroblast adhesion to the ECM is essential for cell spreading, migration, and matrix remodeling during skin repair. ECM components such as FN and COL serve as adhesion substrates and regulate fibroblast behaviors including adhesion, chemotaxis, and migration through interactions with cell surface receptors [14,26]. Studies have shown that recombinant humanized FN (rhFN) and recombinant humanized COL (rhC) enhance fibroblasts’ proliferation, migration, and adhesion, while also improving wound re-epithelialization and collagen deposition [26,29,30]. Therefore, this study employed culture plates coated with rhFN or rhC to evaluate the effect of PDI on HSF cell adhesion. Adhesion was assessed 5 h post-seeding to capture initial attachment while minimizing interference from cell proliferation and autocrine ECM secretion, which become more prominent at later time points.

**Figure 5:**
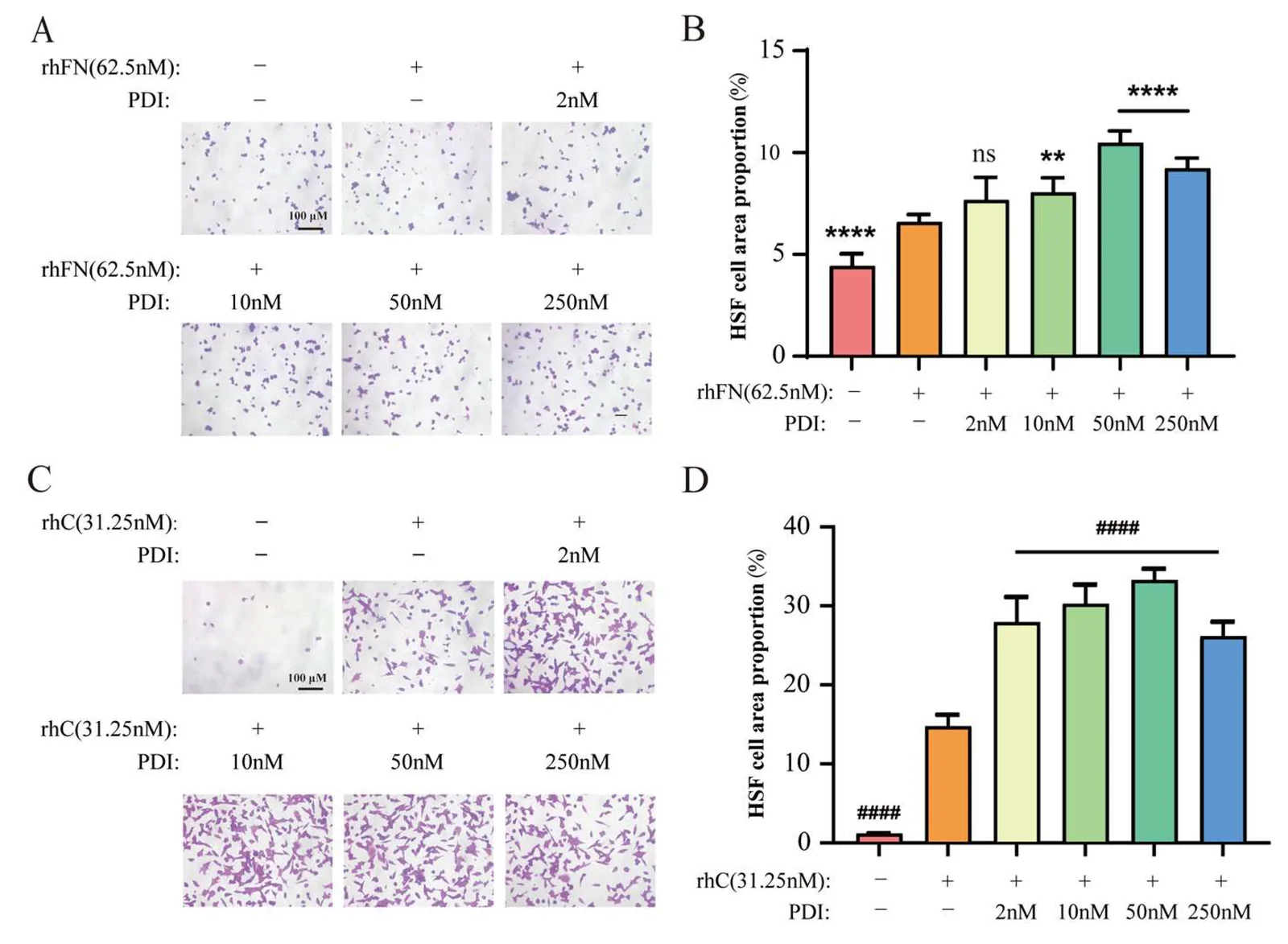
Analysis of the adhesion ability of recombinant PDI protein to HSF cells. (**A**) Images of HSF cell adhesion with PDI combined with rhFN (200×); (**B**) enhancement of HSF cell adhesion to rhFN by PDI; (**C**) images of HSF cell adhesion with PDI combined with rhC (200×); (**D**) enhancement of HSF cell adhesion to rhC by PDI; *n* = 5. Data are expressed as mean ± SD and were analyzed using one-way ANOVA; ** *p* < 0.01, **** *p* < 0.0001, ns: no significant difference compared with the rhFN-alone group; #### *p* < 0.0001 compared with the rhC-alone group.

As shown in Figure 5A,B, under rhFN-coating conditions, the combined use of rhFN and PDI at concentrations of 10–250 nM significantly increased both the number of HSF cell adhesions and the adhesion area compared to the rhFN-alone group (*p* < 0.0001). Similarly, as illustrated in Figure 5C,D, under rhC-coating conditions, co-treatment with recombinant PDI at concentrations of 2–250 nM also markedly enhanced both the number and area of HSF cell adhesions compared to the rhC-alone group (*p* < 0.0001). These results demonstrate that recombinant PDI enhances the adhesion capacity of HSF cells on interfaces coated with either rhFN or rhC. The PDI doses (2–250 nM) used in Figure 5 fall within the non-cytotoxic range defined in Figure 4.

Notably, when directly comparing the two coating conditions, rhC exhibited a superior synergistic effect with PDI over rhFN. The effective concentration threshold for PDI-induced adhesion enhancement was lower on rhC (2 nM) than on rhFN (10 nM), and the relative increases in both adherent cell number and spreading area were consistently more pronounced in the rhC group. This difference suggests that rhC provides a more favorable substrate for PDI-mediated adhesion enhancement, possibly due to the native triple-helical structure of collagen, which better mimics the physiological dermal ECM and supports more robust cell–matrix interactions. In contrast, FN, while also supporting adhesion, may offer a less potent microenvironment for the synergistic action of PDI under the present experimental conditions.

Given PDI’s role in disulfide bond rearrangement and protein conformation regulation, it is hypothesized that exogenous PDI may enhance HSF-ECM adhesion through modulation of ECM protein conformation and/or cell surface sulfhydryl/disulfide status. However, the precise molecular target—whether integrins themselves, ECM proteins, or both—remains to be elucidated; this interpretation should therefore be considered a working hypothesis rather than a concluded mechanism. Future studies employing integrin-blocking antibodies, surface thiol labeling, or site-directed mutagenesis will be necessary for validation.

From the perspective of skin-repair applications, enhanced fibroblast adhesion—quantified here by adherent cell number and spreading area—not only facilitates subsequent proliferation, migration, and matrix deposition but also initiates integrin-mediated signaling that guides regenerative outcomes; while appropriate adhesion promotes healing, excessive or dysregulated adhesive signaling may contribute to fibrosis and scar hypertrophy. Recent studies have also demonstrated that ECM derived from dermal fibroblasts can synergistically promote re-epithelialization and skin tissue repair in collaboration with keratinocytes [31]. Therefore, the adhesion-enhancing effects of recombinant PDI observed at both rhFN and rhC interfaces in this study provide preliminary experimental evidence for its use as a novel active ingredient in skin-repairing cosmeceuticals. However, these findings remain limited to in vitro cellular-level data and cannot be directly generalized to full-scale in vivo skin-repair efficacy. Direct quantification of fibronectin and collagen secretion (e.g., ELISA or immunofluorescence) would also be valuable to confirm whether PDI upregulates ECM protein production. Further validation of its applicability in skin-repair scenarios is required through additional assays, including proliferation assays, cell scratch tests, Transwell migration assays, collagen gel contraction tests, quantification of ECM protein deposition (e.g., fibronectin and collagen secretion via ELISA or immunofluorescence), 3D skin models, and formulation stability evaluations.

## 4. Conclusions

In this study, we successfully produced bioactive recombinant PDI using the Pichia pastoris secretion system. Although PDI has been extensively studied in various biological contexts, its application in skin repair—particularly concerning fibroblast–ECM adhesion—remains underexplored. The purified protein (55.7 kDa) demonstrated no cytotoxicity toward HSF cells and significantly enhanced cell adhesion on fibronectin- and collagen-coated interfaces at low nanomolar concentrations. These findings provide preliminary in vitro evidence supporting the potential of recombinant PDI as a functional ingredient in skin-repair formulations. However, we acknowledge several limitations: the study was confined to short-term adhesion assays, the precise mechanism remains hypothetical, and in vivo efficacy remains untested. Further investigations using 3D skin equivalents and animal wound models are warranted to assess translational feasibility.

## Figures and Tables

**Table 1:** Primers used in the PCR procedure.

Primer	Sequence (5′-3′):
PDI	Upstream primer GAATTCATGGACGCCCCCG
	Downstream primer TGTTCTAGAAAGCTGGCG

## Data Availability

The original contributions presented in this study are included in the article/Supplementary Materials. Further inquiries can be directed to the corresponding author(s).

## Supplementary Materials

The following supporting information can be downloaded at https://www.mdpi.com/article/10.3390/bioengineering13091032/s1. Figure S1. Protein disulfide isomerase titration.

## Abbreviations

The following abbreviations are used in this manuscript:

PDI	Protein Disulfide Isomerase
HSF	Human Skin Fibroblast
rhFN	Recombinant Human Fibronectin
rhC	Recombinant Human Collagen
ECM	Extracellular Matrix
UPR	Unfolded Protein Response
ECL	Enhanced Chemiluminescence
CCK-8	Cell Counting Kit-8
PBS	Phosphate-Buffered Saline
OD	Optical Density
PCR	Polymerase Chain Reaction
DNA	Deoxyribonucleic Acid
BCA	Bicinchoninic Acid (assay)
HRP	Horseradish Peroxidase
IgG	Immunoglobulin G

## References

[1] Baker P., Huang C., Radi R., Moll S.B., Jules E., Arbiser J.L. (2023). Skin barrier function: The interplay of physical, chemical, and immunologic properties. Int. J. Mol. Sci..

[2] Potekaev N.N., Borzykh O.B., Medvedev G.V., Pushkin D.V., Petrova M.M., Petrov A.V., Dmitrenko D.V., Karpova E.I., Demina O.M., Shnayder N.A. (2021). The role of extracellular matrix in skin wound healing. J. Clin. Med..

[3] Wang Y., Zhang Q., Zhou X., Yang D., Xiao L., Xie W., Zheng H., Ye S., Deng C., Cheng Y. (2025). Applications of Fibronectin in Biomedicine and Cosmetics: A Review. Bioengineering.

[4] Xie W., Wu Q., Kuang Z., Cong J., Zhang Q., Huang Y., Su Z., Xiang Q. (2023). Temperature-controlled expression of a recombinant human-like collagen I peptide in *Escherichia coli*. Bioengineering.

[5] Chen X., Shi C., He M., Xiong S., Xia X. (2023). Endoplasmic reticulum stress: Molecular mechanism and therapeutic targets. Signal Transduct. Target. Ther..

[6] Chen X., Cubillos-Ruiz J.R. (2021). Endoplasmic reticulum stress signals in the tumour and its microenvironment. Nat. Rev. Cancer.

[7] Diller R.B., Tabor A.J. (2022). The role of the extracellular matrix in wound healing: A Review. Biomimetics.

[8] Hunt M., Torres M., Bachar-Wikstrom E., Wikstrom J.D. (2024). Cellular and molecular roles of reactive oxygen species in wound healing. Commun. Biol..

[9] Boraldi F., Lofaro F.D., Bonacorsi S., Mazzilli A., Garcia-Fernandez M., Quaglino D. (2024). The role of fibroblasts in skin homeostasis and repair. Biomedicines.

[10] Medinas D.B., Rozas P., Hetz C. (2022). Critical roles of protein disulfide isomerases in balancing proteostasis in the nervous system. J. Biol. Chem..

[11] Murthy A.V., Sulu R., Lebedev A., Salo A.M., Korhonen K., Venkatesan R., Tu H., Bergmann U., Jänis J., Laitaoja M. (2022). Crystal structure of the collagen prolyl 4-hydroxylase (C-P4H) catalytic domain complexed with PDI: Toward a model of the C-P4H α2β2 tetramer. J. Biol. Chem..

[12] Guangzhou Jinan University Medical Biotechnology Research and Development Center, Guangzhou Jiyuan Biotechnology Co., Ltd. Method for expressing recombinant human prolyl-4-hydroxylase in *Pichia pastoris* system and application thereof. CN Patent CN110172470A, 27 August 2019.

[13] Cheng Y., Shi W., Xiao X., Zhang Q., Zhang Q., Su Z., Xiang Q., Huang Y. (2020). New strategy for expression of recombinant human prolyl-4-hydroxylase in *Pichia pastoris*. Pak. J. Zool..

[14] Rahman N.S.A., Zahari S., Syafruddin S.E., Firdaus-Raih M., Low T.Y., Mohtar M.A. (2022). Functions and mechanisms of protein disulfide isomerase family in cancer emergence. Cell Biosci..

[15] Rosenberg N., Mor-Cohen R., Sheptovitsky V.H., Romanenco O., Hess O., Lahav J. (2019). Integrin-mediated cell adhesion requires extracellular disulfide exchange regulated by protein disulfide isomerase. Exp. Cell Res..

[16] Hellewell A.L., Heesom K.J., Jepson M.A., Adams J.C. (2022). PDIA3/ERp57 promotes a matrix-rich secretome that stimulates fibroblast adhesion through CCN2. Am. J. Physiol.-Cell Physiol..

[17] Rellmann Y., Eidhof E., Hansen U., Schulte S., Stücker S., Pap T., Dreier R. (2025). Extracellular ERp57 promotes fibronectin fibril formation during matrix assembly of articular cartilage. Science.

[18] Weiß R.G., Losfeld M.E., Aebi M., Riniker S. (2021). N-Glycosylation Enhances Conformational Flexibility of Protein Disulfide Isomerase Revealed by Microsecond Molecular Dynamics and Markov State Modeling. J. Phys. Chem. B.

[19] Rigobello M.P., Donella-Deana A., Cesaro L., Bindoli A. (2000). Isolation, purification, and characterization of a rat liver mitochondrial protein disulfide isomerase. Free Radic. Biol. Med..

[20] Jayakrishnan A., Wan Rosli W.R., Tahir A.R.M., Razak F.S.A., Kee P.E., Ng H.S., Chew Y.L., Lee S.K., Ramasamy M., Tan C.S. (2024). Evolving paradigms of recombinant protein production in pharmaceutical industry: A rigorous review. Sci.

[21] Charriere G., Bejot M., Schnitzler L., Ville G., Hartmann D.J. (1989). Reactions to a bovine collagen implant: Clinical and immunologic study in 705 patients. J. Am. Acad. Dermatol..

[22] Wu K.J., Wang C.Y., Lu H.K. (2004). Effect of glutaraldehyde on the humoral immunogenicity and structure of porcine dermal collagen membranes. Arch. Oral Biol..

[23] Barone G.D., Emmerstorfer-Augustin A., Biundo A., Pisano I., Coccetti P., Mapelli V., Camattari A. (2023). Industrial production of proteins with *Pichia pastoris*—*Komagataella phaffii*. Biomolecules.

[24] Kosykh A.V., Tereshina M.B., Gurskaya N.G. (2023). Potential role of AGR2 for mammalian skin wound healing. Int. J. Mol. Sci..

[25] Pan Y., Yang J., Wu J., Yang L., Fang H. (2022). Current advances of *Pichia pastoris* as cell factories for production of recombinant proteins. Front. Microbiol..

[26] Dong Y., Zhu W., Lei X., Luo X., Xiang Q., Zhu X., Pan Q., Jin P., Cheng B. (2022). Treatment of acute wounds with recombinant human-like collagen and recombinant human-like fibronectin in C57BL/6 mice individually or in combination. Front. Bioeng. Biotechnol..

[27] Wang Y., Zhang Q., Liao H., Lu X., Cong J., Liu Z., Liu Q., Deng C., Cheng Y., Shu P. (2025). Anti photoaging mechanism of a novel recombinant human fibronectin peptide (rhFNP) derived from the extracellular matrix. Heliyon.

[28] Xie W., Wu Q., Kuang Z., Cong J., Zhang Q., Huang Y., Su Z., Xiang Q. (2023). Temperature-controlled expression of a recombinant human-like collagen I peptide in Escherichia coli. Bioengineering.

[29] Cong J., Cheng Y., Liu T., Cai X., Xu J., Guo R., He R., Xiang Q. (2025). Recombinant human fibronectin segment (rhFN1024) hydrogel carried hPDLSCs to repair diabetic trauma by activated NF-κB signaling pathway. Regen. Biomater..

[30] Cheng Y., Li Y., Huang S., Yu F., Bei Y., Zhang Y., Tang J., Huang Y., Xiang Q. (2020). Hybrid freeze-dried dressings composed of epidermal growth factor and recombinant human-like collagen enhance cutaneous wound healing in rats. Front. Bioeng. Biotechnol..

[31] Dong X., Xiang H., Li J., Hao A., Wang H., Gou Y., Li A., Rahaman S., Qiu Y., Li J. (2025). Dermal fibroblast-derived extracellular matrix (ECM) synergizes with keratinocytes in promoting re-epithelization and scarless healing of skin wounds: Towards optimized skin tissue engineering. Bioact. Mater..

