# Supplementary material for: Cytological Performance Evaluation of a Novel Skin-Repair Agent: Recombinant Protein Disulfide Bond Isomerase
Source: Bioengineering (Basel). 2026 Sep 5;13(9):1032. doi: 10.3390/bioengineering13091032 (PMC13603348; doi:10.3390/bioengineering13091032)
Supplement: Supplementary file 1 [file bioengineering-13-01032-s001.zip › bioengineering-4416344-supplementary.pdf]

Supplementary Materials:

1. Protein disulfide isomerase titration.

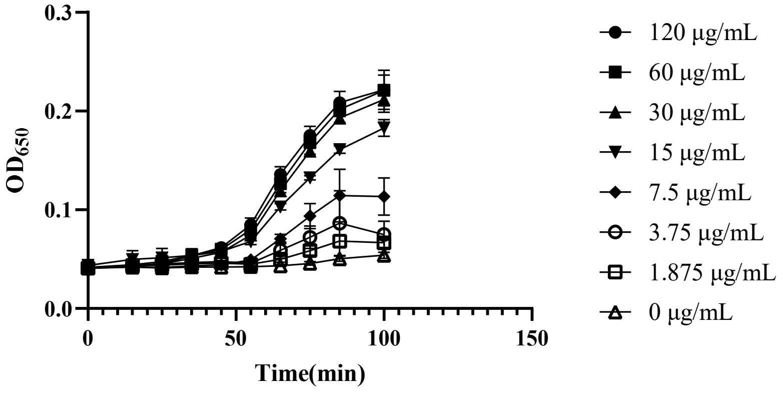

Figure S1. Protein disulfide isomerase titration. Reactions were per-formed according to Materials and Methods with varying PDI concentra-tions (g/mL) as indicated. Absorbance at 650 nm (OD650) was measuredin 5min increments at room temperature. Data are presented as mean +standard deviation (n = 4).
